# New approaches to achieve high level enzyme production in *Streptomyces lividans*

**DOI:** 10.1186/s12934-016-0425-7

**Published:** 2016-02-04

**Authors:** Laura Sevillano, Erik Vijgenboom, Gilles P. van Wezel, Margarita Díaz, Ramón I. Santamaría

**Affiliations:** Instituto de Biología Funcional y Genómica/Departamento de Microbiología y Genética, Consejo Superior de Investigaciones Científicas (CSIC)/Universidad de Salamanca, C/Zacarías González nº 2, 37007 Salamanca, Spain; Molecular Biotechnology, IBL, Sylvius Laboratory, Leiden University, Leiden, The Netherlands

**Keywords:** *Streptomyces*, Protein production, Xylanase, Amylase, Laccase, Vector optimization

## Abstract

**Background:**

Actinomycetes are saprophytic soil bacteria, and a rich source of industrial enzymes. While some of these enzymes can be produced using well-characterized production platforms such as *Escherichia coli* or *Bacillus subtilis,Streptomyces lividans* may be the preferred host for proper folding and efficient secretion of active enzymes. A combination of promoters, signal peptides and hosts were tested in order to obtain the best protein expression in this actinomycete. The xylanase, Xys1, from *S. halstedii*, the α-amylase, Amy, from *S. griseus* and the small laccase, SLAC, from *S. coelicolor* were used as reporters.

**Results:**

The promoters *xysA*p from *S. halstedii* JM8 and *pstS*p from *S. lividans* were the most efficient among those tested. An improvement of 17 % was obtained in xylanase activity when the signal peptide of the α-amylase protein (Amy) of *S. griseus* IMRU3570 was used to direct its secretion. Enhanced expression of SsgA, a protein that plays a role in processes that require cell-wall remodelling, resulted in a improvement of 40 and 70 % of xylanase and amylase production, respectively. Deletion of genes *SLI7232* and *SLI4452* encoding putative repressors of *xysA*p provided improvement of production up to 70 % in the *SLI7232* deletion strain. However, full derepression of this promoter activity was not obtained under the conditions assayed.

**Conclusions:**

*Streptomyces lividans* is a frequently used platform for industrial enzyme production and a rational strain-development approach delivered significant improvement of protein production by this host.

**Electronic supplementary material:**

The online version of this article (doi:10.1186/s12934-016-0425-7) contains supplementary material, which is available to authorized users.

## Background

The production of proteins of industrial interest at low cost is one of the main goals of biotechnology. *Escherichia coli* has usually been a preferred host for protein production, for reasons of high expression levels and short fermentation time. However, frequently insoluble inclusion bodies are formed, which frustrates downstream processing [1–3]. In such cases, alternative production platforms are required, and microorganisms of the genus *Streptomyces* have been successfully applied as a good alternative expression platform for heterologous protein production [4–10]. Streptomycetes are aerobic, filamentous Gram-positive soil bacteria that secrete a wide range of extracellular enzymes, which they require to be able to feast on natural polymers such as starch, cellulose, chitin, mannan or xylan. Their use as a production hosts could overcome some of the problems encountered with other systems: streptomycetes do not readily form inclusion bodies, they have a relatively low level of extracellular protease activity, are well suited to expressing GC-rich genes, and have a high secretion capacity. These properties are very useful for directing the expressed protein in a soluble configuration to the culture supernatant, a major advantage in terms of downstream processing.

Nevertheless, the mycelial lifestyle of actinomycetes results in heteromorphous and viscous cultures, which are unfavourable for industrial fermentation, due to mass-related mechanical stress, heat transfer problems and oxidative stress [11]. Enhanced fragmentation of the mycelia has a major impact on growth and product formation by these organisms and is therefore expected to have an impact on biotechnological applications requiring *Streptomyces* as the production host [12, 13]. The morphology of liquid-grown mycelia is dictated by external factors (media and fermentation conditions), but also by genetic factors. An important example in this respect is the effect of SsgA, which controls all processes that require remodelling of the cell wall, such as germination, tip growth, branching and septum formation [14, 15]. Overexpression of SsgA not only leads to fragmentation of the mycelia, but also to enhanced secretion, with almost three-fold increase of tyrosinase production by *Streptomyces lividans* during batch fermentation [16].

In this work, we show that selection of strong promoters, modification of signal peptides and use of different *S. lividans* hosts result in improved production of putative industrial proteins such as the xylanase Xys1 from *S. halstedii* JM8, the α-amylase Amy from *S. griseus* IMRU3570 and the small laccase SLAC from *S. coelicolor*.

In addition, two transcriptional repressors, XlnR and BlxR, have been identified in *S. lividans* that take part in the control of the *xysA* promoter activity. This provides new handles for the application and further development of the *xysA* promoter as a tool in enzyme production.

## Results

### Promoter choice has a drastic effect on protein expression

In order to find suitable promoters to express proteins in *Streptomyces* we tested six *Streptomyces* promoters for their ability to enable strong gene expression. These promoters are active during different phases of the developmental cycle or regulated by different carbon sources. Two of these were strong promoters used frequently in other studies to express proteins in *Streptomyces*: *vsi*p from *S. venezuelae* [17] and ermE*p from *Saccharopolyspora erythraea* [18], the activity of which was compared to that of promoters of four highly expressed genes that are studied extensively in our laboratory. These promoters are the *xysA*p promoter belonging to the *xysA* gene from *S. halstedii* JM8 that encodes the xylanase Xys1 (U41627) [19], the *pstS* promoter (*pstSp*) from *S. lividans* (AJ698727), which drives transcription of the high-affinity phosphate-binding protein (PstS) [20], and the promoter *xylA*p of *xylA* (SCO1169, encoding xylose isomerase) and *glpQp* of *glpQ* (SCO1968, encoding a glycerophosphoryl diester phosphodiesterase) from *S. coelicolor*.

**Fig. 1:**
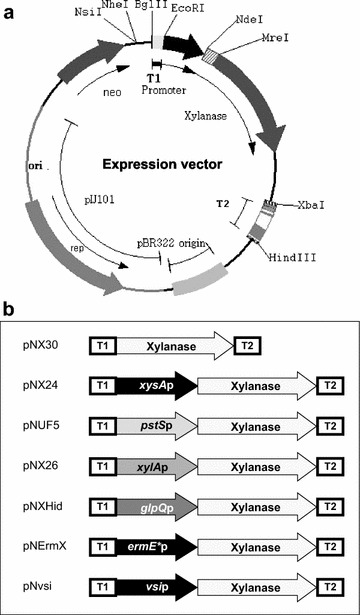
Schematic representation of the plasmids obtained. **a**. General diagram of expression plasmids. The plasmid has the *E. coli* pBR322 origin and the replication region for *Streptomyces* from pIJ101. **b**. Different plasmids with the promoters tested. pNX30 is the control plasmid with a promoterless xylanase gene. pNX24, *xysAp*: promoter of *xysA* gene from *S. halstedii* (U41627); pNUF5, *pstSp*: promoter of the *pstS* gene from *S. lividans* (AJ698727); pNX26, *xylAp*: xylose isomerase promoter from *S. coelicolor* (*SCO1169*); pNXHid, *glpQp*: promoter of a putative hydrolase from *S. coelicolor* (*SCO1968*); pNErmX, ermE*p: promoter of the erythromycin resistance *ermE* gene; pNvsi, *vsip*: promoter of the subtilisin inhibitor SSI from *S. venezuelae* (*X98019*); T1 and T2 are *mmrt* and *fdt* transcriptional terminators

The different promoter sequences were inserted upstream of the xylanase gene *xysA*, which was cloned in a multicopy derivative of the bifunctional *Streptomyces* plasmid pN702GEM3 [21] (see “Methods”). A diagram of the plasmid and of the different constructs is presented in Fig. 1a, b. The constructs pNX24 (*xysA*p) [22], pNUF5 (*pstS*p) [20], pNX26 (*xylA*p), and pNXHid (*glpQ*p), pNErmX (ermE*p), pNvsi (*vsi*p) as well as the promoterless control plasmid pNX30 were introduced into *S. lividans* 1326 by protoplast transformation. Transformants were cultured at 28 °C in liquid YES medium with 1 % xylose or YE with 5 %fructose, as indicated in the Methods section and xylanase production was analyzed by SDS-PAGE of culture supernatants after 3, 5 and 7 days. Extracellular processing results in two bands for xylanase, Xys1L and Xys1S, with a mass of 43.5 and 33.7 kDa respectively [19]. The Xys1S species has lost the C-terminal CBM2 domain but both xylanase species have similar specific activity with oat spelt xylan as substrate [19, 21].

**Fig. 2:**
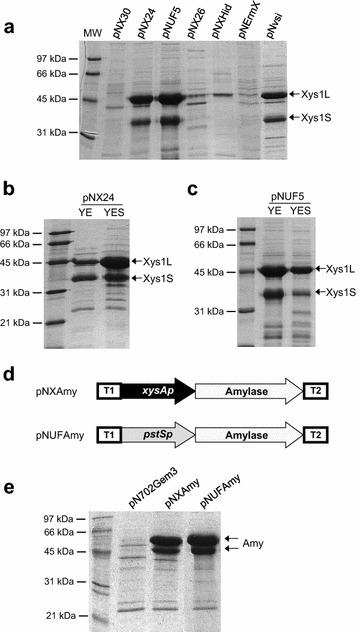
Xylanase and amylase production under the control of different promoters. **a**. Xylanase production by *S. lividans* 1326 transformed with different constructions (pNX30, pNX24, pNUF5, pNX26, pNXHid, pNErmX, and pNvsi) after 5 days of culture in YES medium supplemented with 1 % xylose. **b**. Xylanase production by *S. lividans* 1326 transformed with pNX24 in YE or YES media, both supplemented with 1 % xylose. **c**. Xylanase production by *S. lividans* 1326 transformed with pNUF5 in YE or YES media, both supplemented with 5 % fructose. **d**. Diagram of expression plasmids pNXAmy and pNUFAmy. **e**. Amylase production by *S. lividans* 1326 transformed with pNXAmy in YES medium supplemented with 1 % xylose and with pNUFAmy in YE medium supplemented with 5 % fructose. 10 µL of supernatant of 5 days cultures were loaded in each track. *Arrows* indicate the two bands of 57 and 50 kDa of amylase generated by an intracellular processing [45]

The natural xylanase promoter, *xysA*p, and the *pstS* promoter, *pstS*p, resulted in the highest xylanase production (Fig. 2a lanes 2 and 3). The xylanase production under the control of ermE*p was poor (Fig. 2a lane 6) and, although production under the control of *vsi*p was high, this was lower than that obtained with *xysA*p and *pstS*p (Fig. 2a lane 7 *versus* lanes 2 and 3). Maximum xylanase levels were reached after 5 days, the production did not increase by longer incubation (data not shown).

The production of xylanase under control of *xysA*p and *pstS*p was strongly dependent on the culture medium. For *xysA*p (pNX24) the highest protein production was obtained in YES medium supplemented with 1 % xylose (Fig. 2b), while for *pstS*p (pNUF5) the highest production was obtained in YE medium in the presence of 5 % of fructose [20] (Fig. 2c).

To demonstrate that *xysA*p and *pstS*p are generally applicable for enzyme production, they were used to drive the expression of α-amylase (Amy) from *S.griseus* obtaining the plasmids pNXAmy and pNUFAmy respectively (Fig. 2d). High production levels of this enzyme were also obtained demonstrating the potential of these promoters to express other proteins of interest (Fig. 2e).

### Modification of the secretion signal peptide improves protein export

**Fig. 3:**
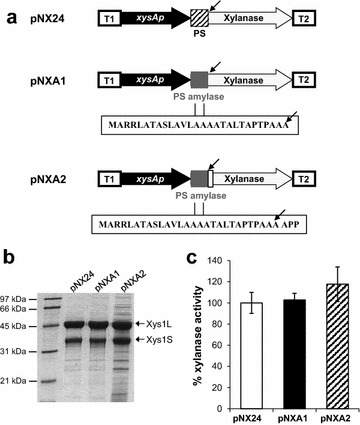
Signal peptide modifications and its effect. **a**. Diagram of the modifications in the xylanase signal peptide. The *arrows* indicate the processing point of the signal peptide. T1 and T2 are *mmrt* and *fdt* transcriptional terminators. (PS: signal peptide) **b**. Xylanase production by *S. lividans* 1326 transformed with pNX24, pNXA1, or pNXA2 after 5 days of culture in YES supplemented with 1 % xylose. 10 µL of supernatant were loaded in each track. **c**. Xylanase activity of supernatants. The histograms are the means of three different experiments

Changes in the signal peptide might be advantageous for secretion efficiency and therefore for increasing the production of heterologous proteins [23, 24]. In order to compare the efficiency of the α-amylase (Amy) signal peptide with that of xylanase (Xys1), we replaced the codons for the Xys1 signal peptide in pNX24 (first 45 codons) by those for the corresponding Amy signal peptide (28 codons) [25], resulting in pNXA1 (Table 1, Fig. 3a). A xylanase derivative with the complete Amy signal peptide coding sequence including the first three codons of the mature amylase was also constructed in order to maintain the environment of the amylase signal peptide-cleavage site (pNXA2, Fig. 3a).

**Table 1:** Plasmids used in this work

Plasmid	Characteristics	Reference
pN702GEM3	*E. coli*–*Streptomyces* shuttle vector; contains the pIJ702 origin of replication (for *Streptomyces*), the pUC origin of replication (for *E. coli*) and a polylinker derived from pGEM3Zf(+) (Promega). It carries the bifunctional Neo/Kan resistance marker from Tn5 (for both *E. coli* and *Streptomyces*)	[21]
pNX30	pN702GEM3 derivative. *xysA* xylanase ORF without any promoter and flanked by transcriptional terminators. Used as negative control	[35]
pNX24	pN702GEM3 derivative. *xysA* promoter from *S. halstedii* controls xylanase expression	[22]
pNUF5	pN702GEM3 derivative. *pstS* promoter from *S. lividans* controls xylanase expression	[20]
pNErmX	pN702GEM3 derivative. Erythromycin resistance promoter from *S.* *erythraeus* controls xylanase expression	This work
pNvsi	pN702GEM3 derivative. *vsi* promoter from *S. venezuelae* controls xylanase expression	This work
pNX26	pN702GEM3 derivative. Xylose isomerase promoter from *S. coelicolor* controls xylanase expression	This work
pNXHid	pN702GEM3 derivate. Promoter of a putative hydrolase (*glpQ*) from *S. coelicolor* controls xylanase expression	This work
pNXA1	pNX24 derivate. *xysA* promoter from *S. halstedii* controls xylanase expression with amylase signal peptide	This work
pNXA2	pNX24 derivative. *xysA* promoter from *S. halstedii*. Controls xylanase expression with the amylase signal peptide with three additional amino acids	This work
pNXAmy	pN702GEM3 derivative. *xysA* promoter from *S. halstedii* controls amylase expression	This work
pNUFAmy	pN702GEM3 derivative. *pstS* promoter from *S. lividans* controls amylase expression	This work
pEPOS101	pHJL401 derivative harbouring *xysA*p driving the expression of SLAC (*SCO6712*)	This work

Cultures of *S. lividans* harbouring either pNX24 or its derivatives pNXA1 or pNXA2 were grown at 28 °C for 5 days in liquid YES medium supplemented with 1 % xylose; the amount of xylanase in the supernatant was analyzed by SDS-PAGE, xylanase bands intensities were determined with the ImageJ software and the activity quantified using the DNS assay (“Methods”) (Fig. 3b, c and Additional file 1: Tables S1, S2). Replacing the original xylanase signal peptide with that of amylase resulted in a similar degree of xylanase secretion (pNXA1, Fig. 3c). However, an increase of 17 % in xylanase activity was obtained when the amylase signal peptide with the three aminoacids extension was used (pNXA2) (Fig. 3c and Additional file 1:Table S2).

### Engineered hosts for improved protein yield

The host used as platform for enzyme production is a major determinant for the yield that can be obtained. We explored two ways of improving the host: (1) the effect of host morphology on xylanase production and (2) the effect of putative repressors on *xysA* expression. To change the morphology we selected a *S. lividans* 1326 strain overexpressing SsgA (GSAL1) [16]. The *ssgA* gene is a morphogene that pleiotropically affects growth and cell division [26]; it activates sporulation-specific cell division by ensuring the correct localization of SsgB, which in turn recruits FtsZ to future septum sites [27]. The enhanced expression of *ssgA* leads to strongly enhanced septation in vegetative hyphae, which results in fragmented growth and much wider hyphae, a phenotype that favours protein production as well as secretion [16].

**Fig. 4:**
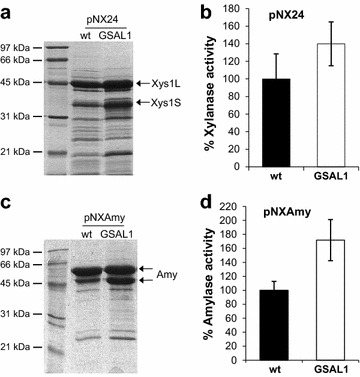
SsgA effect on xylanase and amylase production. **a**. Xylanase production by *S. lividans* 1326 (wt) and the SsgA overproducer strain (GSAL1), transformed with pNX24 after 5 days of culture in YES supplemented with 1 % xylose. **b**. Percentage of xylanase activity of supernatants. C. Amylase production by *S. lividans* 1326 (wt) and the SsgA overproducer strain (GSAL1) transformed with pNAmy after 5 days of culture in YES supplemented with 1 % xylose. **d**. Percentage of amylase activity of supernatants. The histograms are the mean of three different experiments

In order to establish the effect of SsgA overexpression on xylanase production, pNX24 was introduced to *S. lividans* 1326 and its derivative overexpressing SsgA (GSAL1). The transformants were grown for 5 days in YES medium with 1 % xylose and the amount of xylanase in the culture supernatants was analysed by SDS-PAGE (Fig. 4a) and the xylanase activity was quantified by the DNS protocol (Fig. 4b). Both, the total amount of xylanase (Xys1L + Xys1S) bands, as determined with ImageJ, and the enzyme activity increased in the SsgA-overexpressing host GSAL1. The band intensities in the SDS-PAGE increased by 70 % while the enzyme activity increased by 40 % (Fig. 4a, b and Additional file 1:Tables S3, S4).

*Streptomyces lividans* 1326 and GSAL1 were also transformed with pNXAmy harbouring the α-amylase gene under control of *xysAp*, and the culture supernatants were analysed by SDS-PAGE (Fig. 4c) and the amylase activity of the samples was quantified by the DNS protocol (Fig. 4d). The GSAL1 transformant showed an increase of 45 % in the band intensities while the amylase activity increased by 70 % (Fig. 4c, d and Additional file 1:Tables S5 and S6). Both strains were also transformed with the empty plasmid (pN702GEM3) as a control showing no detectable xylanase or amylase activity in the quantification assays (data not shown).

The second approach was prompted by the observation that maximum enzyme production, controlled by *xysAp,* was obtained after 4 days of growth in liquid media with low accumulation after 24 h (data not shown). This suggests that a repressor(s) bound to *xysA*p might be controlling its expression during the first days of growth. Carbon catabolite repression of *xysAp* was demonstrated previously and the role of different regions of the promoter studied [28]. Among them, a motif 5′-CGAAACTTTCG-3′, also present in promoter regions of a number of *S. lividans* genes, all of them related to xylan metabolism (data not shown), seems to be important in the glucose control of this promoter [28]. This palindromic sequence is the binding site for BxlR, a xylobiose responsive repressor in *S. thermoviolaceus* [29]. The homologue of BxlR in *S. lividans* is encoded by SLI7232. In addition to BxlR, another putative repressor, XlnR (SLI4215) has been implicated in xylanase expression in *S. lividans* TK24 [30].

In order to test whether these two repressors may affect the temporal accumulation pattern of an enzyme which gene is controlled by *xysAp,* the gene for the small laccase, SLAC [31] was used as reporter under the control of the *xysAp* (plasmid pEPOS101). SLAC was used as reporter to eliminate potential interference of the induction of host xylanases and xylose metabolism related enzymes in *bxlR* and *xlnR* deletion strains.

**Fig. 5:**
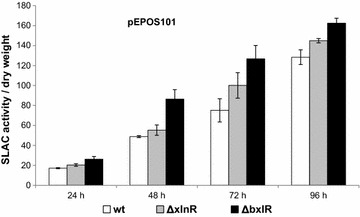
Effect of the deletion of the repressor genes *xlnR* and *bxlR* on the production of SLAC controled by *xysA*p. SLAC activity normalized for biomass was determined in the wild type *S. lividans* 1326, ΔxlnR and ΔbxlR strains with DMPPDA as substrate as described in methods. Three independent transformants of each strain were analysed for SLAC activity in duplicate. Maximum biomass (determined as g/L) was reached around 48 h incubation and remained essentially unchanged up to 96 h

Both repressor mutant strains (obtained as indicated in “Methods”) showed an increased SLAC expression compared to the wild type parent, *S. lividans* 1326. The highest increase was observed in the *∆bxlR* strain with 70 % more SLAC after 72 h of growth and 30 % after 96 h. However, the profile of enzyme activity over time was similar in all three strains and complete derepression was not obtained during the first 2 days of incubation suggesting additional levels of control (Fig. 5).

## Discussion

The high-level production of heterologous proteins is a key objective in biotechnology. *S. lividans* is a preferred heterologous host for industrial production of proteins from prokaryotic and eukaryotic organisms [5, 10, 32]. It has several major advantages over the more traditional platforms such as *E. coli* and *B. subtilis* as well as compared to other *Streptomyces* species. These advantages include: (i) low endogenous extracellular proteolytic activity in comparison with other *Streptomyces* species or *Bacillus* [33, 34]; (ii) the fact that *S. lividans* does not form inclusion bodies, as is often the case in *E. coli*; and (iii) its ability to produce bioactive proteins [5]. Furthermore, the *Streptomyces* protein secretion machinery very efficiently exports proteins of interest into the culture supernatant, which facilitates subsequent protein purification. However, *S. lividans* also has some significant drawbacks, which relate to the mycelial growth associated with slow growth and viscous cultures and the lack of well-established expression systems. Thus, the implementation of *Streptomyces* as a production platform requires the optimization of promoter/vector/host systems.

In this work, we expand the toolbox of available promoters in combination with secretion signals to improve protein production in *S. lividans*. Several strong, constitutive promoters have been previously described for *Streptomyces*, but the number of suitable inducible promoters is scarce [3]. We compared six promoters and obtained good expression of the reporter proteins Xys1 from *S. halstedii* and Amy from *S. griseus* using the promoters *xysA*p, which is induced by different carbon sources such as xylose, xylan and fructose and repressed by glucose [28], and *pstS*p, induced by low phosphate conditions and by different carbon sources such as fructose, xylose or galactose [20, 35, 36]. These promoters resulted in more efficient and reproducible protein production than the widely applied *Streptomyces* promoters ermE*p and *vsi*p (Fig. 2a lanes 2 and 3 versus lanes 6 and 7). Therefore, the high-copy number *E. coli* and *Streptomyces* shuttle plasmids developed in this work; equipped with strongly inducible promoters (*xysA*p or *pstS*p), represent a good option for producing high levels of proteins of interest in *Streptomyce*s as has been demonstrated for the xylanase, (Xys1) from *S. halstedii*, the α-amylase (Amy) from *S. griseus* and laccase (SLAC) from *S. coelicolor*.

The elimination of the putative *xysA*p repressors XlnR or BxlR from the host strains increased the protein production of the reporter SLAC in these mutants (up to 70 % after 72 h in the Δ*bxlR* strain) indicating the suitability of these strains. Moreover, in the Δ*bxlR* strain, the activity is clearly induced earlier with an almost twofold higher SLAC production at the onset of stationary growth at 48 h. The fact that repression is not completely relieved in these single mutants during logarithmic growth (24 h) suggests that multiple (negative) control mechanisms act on *xysAp* in *S. lividans*.

We then proceeded to test the effect of altering the signal peptide, the adaptation of which could be a valuable additional tool in the production process to obtain more efficient secretion. As reviewed by Lammertyn and Anné [23], several modifications have been found that improve *Streptomyces* signal peptides, and some of these were applied successfully in the vectors developed in this study. We observed an improvement of 17 % in xylanase secretion by changing the xylanase signal peptide for an amylase signal peptide with three additional amino acids from the mature amylase to maintain the sequence around the signal peptidase cleavage site (Fig. 3c). However, modification of the net charge of the amylase signal peptide as it has been described in amylase secretion [25] did not further improve xylanase production (data not shown). Thus, changes in the secretion signal that work for one protein do not necessarily also work for others because this effect is highly sequence dependent and may be also affected by the level of expression reached. Additionally, changing the N-terminal part of the gene may also have repercussions at the level of translation.

As discussed above, protein production is not only about providing the optimal expression system, but also depends on the morphology of the production host, which is a major factor in determining production level as well as fermentation time. Problems associated with the fermentation of filamentous organisms include slow growth rates, high viscosity, mixing problems due to the formation of large mycelial clumps and complex downstream processing [16]. Enhanced expression of the SsgA protein in *S. lividans* reduces such problems significantly as it favours fragmented growth due to enhanced cell division [15]. Interestingly, SsgA is also linked to the expression of all of the components of the secretion machinery. For example, enhanced expression of SsgA results in a strong increase in the secretion of tyrosinase (which is a substrate for the Tat system) in much shorter fermentation time, and expression of genes encoding components of the Tat and Sec secretion machinery is strongly upregulated in *ssgA* null mutants [14]. The latter is most likely the result of a feedback response to compensate for the absence of the important morphoprotein SsgA [14]. As a result of the improved growth and secretion capacity, the enhanced expression of SsgA has been shown to be potentially very advantageous for protein production [16]. Indeed, xylanase and amylase production increased by 40 and 70 %, respectively, in *S. lividans* GSAL1, which overexpresses SsgA when compared to the wild type strain (Fig. 4b, d).

## Conclusions

The use of *Streptomyces* as a platform for high-level proteins production requires proper optimization for each protein of interest. We constructed a set of bifunctional plasmids (*E. coli*-*Streptomyces*) with strong and inducible promoters that are convenient for protein production in *Streptomyces*. Combination with hosts with either enhanced expression of SsgA or lacking specific transcriptional repressors further improved enzyme production.

## Methods

### Strains and culture conditions

*Escherichia coli* DH5α [37] was used for the cloning and isolation of plasmids. It was grown in Luria–Bertani liquid broth or on LB agar. DNA manipulations in *E. coli* were done following standard procedures [38].

*Streptomyces lividans* 1326 and its derivative strains, GSAL1 [16], Δ*xlnR,* and Δ*bxlR* were used as hosts. The genome sequence of *S. lividans* 1326 has been published [39] and is available at StrepDB (http://strepdb.streptomyces.org.uk). GSAL1 is a transformant of *S. lividans* harbouring the integrative plasmid pGWS4-SD, which results in the effective overproduction of the SsgA protein [15]. The Δ*xlnR* and Δ*bxlR* mutants were prepared and isolated according to protocols described previously [40]. In the *∆xlnR* mutant, nucleotides −146 to +756 relative to the start codon of *SLI4452* were replaced by a 62 nt scar of the *loxP* recombination site including two XbaI sites. In the *∆bxlR* mutant, nts +1 to +1127 relative to the start of *SLI7232* were replaced. Strains were grown on R2YE agar plates for transformation, on MSA agar plates for sporulation [41] and in YE (1 % (w/v) Yeast extract, 5 mM MgCl_2_ pH 7.2) or YES (1 % (w/v) Yeast extract, 10.3 % (w/v) sucrose, 5 mM MgCl_2_ pH 7.2) liquid media supplemented with 1 % (w/v) of xylose or 5 % (w/v) of fructose for protein expression [20]. Neomycin, 15 μg/mL, or thiostrepton, 2.5 μg/mL, were added to the culture depending of the plasmid used. Liquid-grown cultures were carried out in baffled flasks at 28 °C and 200 rpm. DNA manipulations in *Streptomyces* were carried out essentially as described by Kieser et al. [41].

### Plasmid constructions

All plasmids used in the present work are listed in Table 1. PCR amplification of each promoter was accomplished with oligonucleotides including EcoRI and NdeI restriction sites (Table 2). PCR products were purified by agarose gel electrophoresis, digested with EcoRI and NdeI, and cloned in plasmid pNX30 [5] digested with the same restriction enzymes.

**Table 2:** Oligonucleotides used in this work

Name	Sequence 5′–3′	Use
LS-001	TTTTTT*GAATTC*TGTGCGGCTGCCCTTCCGCC	Forward oligonucleotide for cloning the *glpQ*p. The sequence recognized by EcoRI is in italics
LS-002	TTTTTT*CATATG*CGTACTCCTCGCGTCGAACG	Reverse oligonucleotide for cloning the *glpQ*p. The sequence recognized by NdeI is in italics
LS-Amy	TTTTTT*CATATG*GCCCGCAGACTCGCCACC	Forward oligonucleotide to introduce the amylase signal peptide. The sequence recognized by NdeI is in italics
LS-003	TTTTTT*CGCCGGCG*GCAGCGGCGGGTGTG	Reverse oligonucleotide to introduce the amylase signal peptide. The sequence recognized by MreI is in italics
LS-004	TTTTTT*CGCCGGCG*GGCGGGGCGGCAGCGGC	Reverse oligonucleotide to introduce the amylase signal peptide with three additional amino acids. The sequence recognized by MreI is in italics
LS-023	TTTTTT*GAATTC*GGTACCAGCCCGACCCGAGC	Forward oligonucleotide for cloning the ermE*p. The sequence recognized by EcoRI is in italics
LS-024	TTTTTT*CATATG*ACCAACCGGCACGATTGTGCC	Reverse oligonucleotide for cloning the ermE*p. The sequence recognized by NdeI is in italics
LS-026	TTTTTT*GAATTC*GGGGATGACCACCGCGGGAG	Forward oligonucleotide for cloning the *vsip*. The sequence recognized by EcoRI is in italics
LS-027	TTTTTT*GATATC*GGTGAACTCTCCTTCGATCGATG	Reverse oligonucleotide for cloning the *vsip*. The sequence recognized by EcoRV is in italics
RS-003	TTTTTT*GAATTC*GGGCTTCTCCCTCTTCCGCGGG	Forward oligonucleotide for cloning the *xylp*. The sequence recognized by EcoRI is in italics
RS-004	TTTTTT*CATATG*CCGCGGCTCCTCACTCGCTGC	Reverse oligonucleotide for cloning the *xylp*. The sequence recognized by NdeI is in italics
LS-celF	TTTTTT*GAATTC*GGCCGGCCGCTCCCGTCTGGC	Forward oligonucleotide for cloning the *celAp*. The sequence recognized by EcoRI is in italics
LS-celR	TTTTTT*CATATG*CAGTACCTCGATTTCAGAGGA	Reverse oligonucleotide for cloning the *celAp*. The sequence recognized by NdeI is in italics
MRG11	TTTTTT*CATATG*GCCCGCAGACTCCGCACC	Forward oligonucleotide for cloning amylase ORF. The sequence recognized by NdeI is in italics
MRG12	TTTTTT*CTCGAG*GCCGCGCCAGGTGTCGTTGAG	Reverse oligonucleotide for cloning amylase ORF. The sequence recognized by XhoI is in italics
4215UpF	GCG*GAATTC*AGCTGCTCAAGGACGCCGGC	Forward oligonucleotide for the cloning upstream flank of XlnR. The sequence recognized by EcoRI is in italics
4215UpR	GCG*GGATCC*CATCCCGTGGGCCTCCTCTCC	Reverse oligonucleotide for the cloning upstream flank of XlnR. The sequence recognized by BamHI is in italics
4215DwF	GCG*TCTAGA*GTAACTCGAGCGGTCTCGCCC	Forward oligonucleotide for the cloning downstream flank of XlnR. The sequence recognized by XbaI is in italics
4215DwR	CGC*AAGCTT*CCGCATCTGCTGGAGCCGG	Reverse oligonucleotide for the cloning downstream flank of XlnR. The sequence recognized by HindIII is in italics
7232UpF	GCG*GAATTC*GTGAGGTGTGTGGTCATGAGCC	Forward oligonucleotide for the cloning of upstream flank of BxlR. The sequence recognized by EcoRI is in italics
7232UpR	CGC*TCTAGA*CCGGGCGCCCACCTCAAC	Reverse oligonucleotide for the cloning upstream flank of BxlR. The sequence recognized by XbaI is in italics
7232DwF	GCG*TCTAGA*GGCTCCTACCGGCCGGGC	Forward oligonucleotide for the cloning downstream flank of BxlR. The sequence recognized by XbaI is in italics
7232DwR	CGC*AAGCTT*GTGCCGCGGCCGGAGCCG	Reverse oligonucleotide for the cloning downstream flank of BxlR. The sequence recognized by HindIII is in italics

pNXA1 and pNXA2 were constructed by PCR amplification of the signal peptide of the α-amylase (EMBL X57568) from *S. griseus* IMRU 3570 with oligonucleotides including NdeI and MreI restriction sites (MreI cuts just after the sequence that encodes the signal peptide of the xylanase and the correct frame was conserved in the construction) (Table 2). PCR products were purified by agarose gel electrophoresis, digested with NdeI and MreI and cloned in plasmid pNX24 [22] digested with the same restriction enzymes. The new constructs have the α-amylase signal peptide fused in-frame with the *xysA* gene.

The plasmids pNXAmy and pNUFAmy were constructed by replacing xylanase gene by the α-amylase ORF in pNX24 and pNUF5 respectively. The α-amylase gene (*X57568.1*) was amplified from *S. griseus* genome with oligonucleotides MRG11 and MRG12 (Table 2).

The inserts of all plasmid constructions were sequenced on both strands using a Perkin–Elmer ABI Prism 377 DNA sequencer. *In silico* plasmids were done with the Gene Construction Kit (GCK, Textco).

### Protein analysis

Protein profiles were analysed by denaturing polyacrylamide gel electrophoresis (SDS-PAGE) in a MiniProtean II system (Bio-Rad). Proteins were detected by 0.5 % Coomassie brilliant blue R staining and Low-Range SDS-PAGE Standards (Bio-Rad) were used as markers. Xylanase and amylase band intensities were analysed with ImageJ software [44].

### Xylanase and amylase activities assays

Xylanase and amylase activities were measured by the dinitrosalicylic acid (DNS) method, using xylose or maltose as standards respectively [42]. One unit of xylanase or amylase activities was defined as the amount of enzyme required releasing 1 µmol of reducing sugars, (expressed as xylose equivalents or maltose equivalents), in 1 min. All data shown are means of at least three different experiments.

### SLAC in-gel assay

Secreted SLAC activity was determined in the supernatant of liquid cultures of the wild type *S. lividans* 1326, Δ*xlnR* and Δ*bxlR* strains harbouring the SLAC reporter plasmid pEPOS101. Cells were grown in YES medium supplemented with 1 % xylose, 20 µM Cu(II) and 2.5 µg/mL thiostrepton and samples (1.5 mL) were taken after 24, 48, 72 and 96 h of incubation at 28 °C. In-gel SLAC activity stain was done essentially according to Endo et al. [43]. Samples were mixed 1:1 with SDS-PAGE loading buffer without β-mercaptoethanol and without boiling and loaded directly on the gels. Following electrophoresis and detection of SLAC with N,N-Dimethyl-p-phenylenediamine sulfate salt (DMPPDA, Sigma D-4790) and naphthol, digital images of the gels were taken and analysed with ImageJ [44]. Band intensities were normalized for the biomass (dry weight) and expressed in arbitrary units per dry weight.

## Additional file

10.1186/s12934-016-0425-7 Supplementary tables (S1–S6) containing enzyme quantification by activity or by ImageJ.
