# Supplementary material for: New approaches to achieve high level enzyme production in Streptomyces lividans
Source: Microb Cell Fact. 2016 Feb 4;15:28. doi: 10.1186/s12934-016-0425-7 (PMC4743123; doi:10.1186/s12934-016-0425-7)
Supplement: Supplementary file 1 — 10.1186/s12934-016-0425-7 Supplementary tables (S1–S6) containing enzyme quantification by activity or by ImageJ. [file 12934_2016_425_MOESM1_ESM.docx]

**Table S1:** Quantification of xylanase bands of picture 3B by ImageJ software. The numbers are arbitrary units.

|  | **Xys1L** | **Xys1S** | **Total** |
| --- | --- | --- | --- |
| **pNX24** | 17,4 | 11,8 | 29,2 |
| **pNXA1** | 20,1 | 12,8 | 32,9 |
| **pNXA2** | 21,6 | 16,0 | 37,7 |

**Table S2:** Xylanase activity of supernatants corresponding to Figure 3C. One Unit of Xylanase activity is defined as the amount of enzyme required to release 1 µmol of reducing sugar expressed as xylose equivalents.

|  | **Xylanase activity (U/mL)** | **SE** |
| --- | --- | --- |
| **pNX24** | 182,0 | 18,0 |
| **pNXA1** | 187,2 | 11,3 |
| **pNXA2** | 214,3 | 29,8 |

**Table S3:** Quantification of xylanase bands of picture 4A by ImageJ software. The numbers are arbitrary units.

|  | **Xys1L** | **Xys1S** | **Total** |
| --- | --- | --- | --- |
| **wt pNX24** | 24,8 | 11,8 | 36,7 |
| **GSAL1 pNX24** | 35,1 | 28,0 | 63,2 |

**Table S4:** Xylanase activity of supernatants corresponding to Figure 4B. One Unit of Xylanase activity is defined as the amount of enzyme required to release 1 µmol of reducing sugar expressed as xylose equivalents.

|  | **Xylanase activity (U/mL)** | **SE** |
| --- | --- | --- |
| **wt pNX24** | 178,8 | 29,5 |
| **GSAL1 pNX24** | 250,2 | 25,9 |

**Table S5:** Quantification of amylase bands of picture 4C by ImageJ software. The numbers are arbitrary units.

|  | **Amy1** | **Amy2** | **Total** |
| --- | --- | --- | --- |
| **wt pNXAmy** | 34,0 | 6,7 | 40,8 |
| **GSAL1 pNXAmy** | 35,6 | 23,5 | 59,1 |

**Table S6:** Amylase activity of supernatants corresponding to Figure 4D. One Unit of Amylase activity is defined as the amount of enzyme required to release 1 µmol of reducing sugar expressed as maltose equivalents.

|  | **Amylase activity (U/mL)** | **SE** |
| --- | --- | --- |
| **wt pNXAmy** | 92,7 | 11,7 |
| **GSAL1 pNXAmy** | 159,3 | 27,2 |
